# Effect of the high voltage waveform on the ionic wind produced by a needle-to-plate dielectric barrier discharge

**DOI:** 10.1038/s41598-022-23417-0

**Published:** 2022-11-04

**Authors:** Eric Moreau, Etienne Defoort

**Affiliations:** Pprime Institute, CNRS, ISAE-ENSMA, University of Poitiers, Téléport 2, BP 30179, 86962 Futuroscope, France

**Keywords:** Fluid dynamics, Applied physics

## Abstract

Although corona discharges are used in many industrial applications because of their ability to produce chemical species, the ionic wind they induce is less known and often ignored. Therefore, the present study aims at investigating the ionic wind produced by a corona discharge ignited between a high voltage needle and a grounded plate electrode covered by a dielectric material. More specifically, the work focuses on the influence of the high voltage waveform on the temporal behavior of the ionic wind. The results highlight that the high voltage waveform plays a key role on the dynamics of the flow produced inside the discharge. On the one hand, for the sine, triangle and sawtooth waveforms, there is a flow acceleration during both the positive and the negative half-cycles, the positive discharge being more effective in velocity production. On the other hand, for the square waveform, the increase in velocity occurs during the rises and falls of the voltage, because of the strengthening of the electric field due to the ions remaining from the previous half-cycle at the wall of the dielectric material.

## Introduction

A corona discharge can be generated in air at atmospheric pressure when a sufficiently high voltage is applied on a thin active electrode. In most engineering applications, corona discharges are used to modify the gas chemistry for ozone production^1,2^, reduction of gaseous pollutants^3,4^, surface treatment^5^ or assisted-combustion^6–8^ to name a few examples. They can also be used for their electroaerodynamic properties, either for manipulating the trajectory of particles electrically charged by the discharge in electrostatic precipitators and separators^9–12^, either to induce a thrust and a jet flow in electroaerodynamic propelers^13,14^ or for airflow control^15–17^ and thermal applications^18,19^.

Indeed, the ions produced around the thin electrode are subjected to the Coulomb force, and therefore move towards the grounded electrode. The set of all the Coulomb forces acting on every ion is at the origin of an electroaerodynamic body force that takes place within the discharge. Moreover, the ions drift toward the collecting electrode because the electric field. On their path, they exchange momentum with the surrounding air neutral molecules by collisions, thus inducing a motion of the bulk of fluid (e.g. a jet) from the high voltage electrode towards the collecting electrode. This flow is called the ionic wind. This phenomenon can be used as an electromechanical actuator without moving parts, which directly converts electrical energy into a flow (i.e. mechanical energy).

On the one hand, the time-averaged velocity of the ionic wind produced by DC positive and negative discharges has been widely studied during the last decades^20–23^ and a review paper^24^ has been recently published on this subject. Furthermore, several fundamental studies have been conducted in the last few years on the temporal behavior of the ionic wind produced by DC positive and negative point-to-plate corona discharges, using high frequency particle image velocimetry systems^25–29^. They have allowed a better understanding of the link between the discharge regime and the ionic wind dynamics.

On the other hand, there is no scientific article on the temporal behavior of the ionic wind induced by a corona discharge powered by an AC high voltage, and even less is known on the effect of the waveform of the AC applied voltage on the dynamics of the produced flow.

Therefore, the present study aims at characterizing precisely the time-averaged and time-resolved ionic wind produced by a corona discharge established between a needle powered by AC high voltages with different waveforms (sine, triangle, sawtooth and square) and a grounded plate covered by a dielectric material. The latter allows to stabilize the discharge and to prevent the corona-to-spark transition. The air gap is here fixed at 15 mm.

In a first part of this article, the electrical properties of the point-to-plate dielectric barrier discharge (DBD) powered by different high voltage waveforms are presented. Then, the study focuses on the resulting time-averaged ionic wind and its time-resolved dynamics.

## Results and discussion

### Electrical measurements

**Figure 1:**
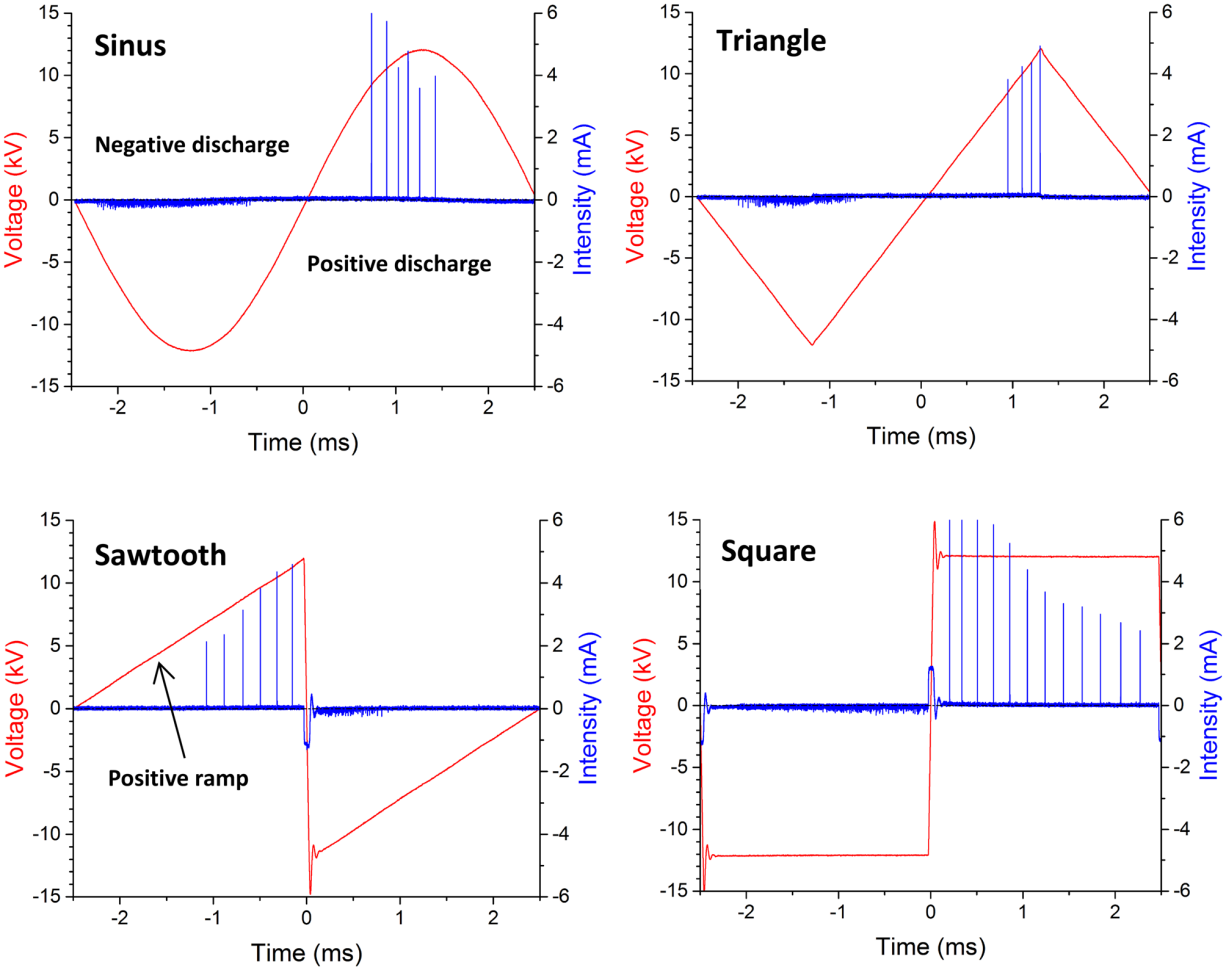
Voltage and discharge current versus time for a voltage amplitude V_AC_ = 12 kV and a voltage frequency f_AC_ = 200 Hz for different voltage waveforms.

Figure 1 presents an example of high voltage v_AC_(t) and discharge current i(t) versus time, for an amplitude value V_AC_ = 12 kV and a frequency f_AC_ = 200 Hz for the four different high voltage waveforms. In all the cases, two different discharges take place, one during the positive half-cycle and another one during the negative-half cycle, such as for surface dielectric barrier discharges used for flow control applications^16^. On the one hand, during the positive half-cycle, a positive streamer corona discharge occurs. This is highlighted by high current pulses with magnitudes reaching about 6 mA here and a frequency varying between 5 and 7 kHz (in the case of the sine high voltage). On the other hand, one can observe that the negative corona discharge results in more numerous current pulses but weaker in magnitude, each of them corresponding to a Trichel pulse, as in negative DC coronas^27^. When focusing on these Trichel pulses, one can see that their amplitude is limited to about 0.2 mA with a frequency between 100 and 400 kHz, depending on the v_AC_(t) value. In the case of the triangle and sawtooth high voltages, the amplitude of the streamers increases during the positive increasing ramp. In fact, as for positive DC coronas, the amplitude of streamers increases with the voltage applied between both electrodes. With the square high voltage, the behavior is radically different. At the beginning of the positive half-cycle (t slightly higher than 0), there are high amplitude streamers (up to 6 mA) with a time interval between two successive streamers of around 130 µs (frequency of about 7 kHz). When t increases, positive ions are accumulated on the dielectric wall, resulting in a diminution of the voltage across the discharge. Therefore, this results in a diminution of the amplitude and frequency of the streamers, as for a DC corona when the DC applied high voltage is decreased^26^.

The last remark concerns the current bump occurring at t = 0 for both the square and sawtooth high voltages (HV). For the sawtooth HV, this current is due to the sudden return of positive ions from the dielectric wall toward the needle because the polarity reversal. Similarly, the same behavior can be observed for the square HV because of the return of negative ions at t = 0 and positive ions at t =  − 2.5 ms.

**Figure 2:**
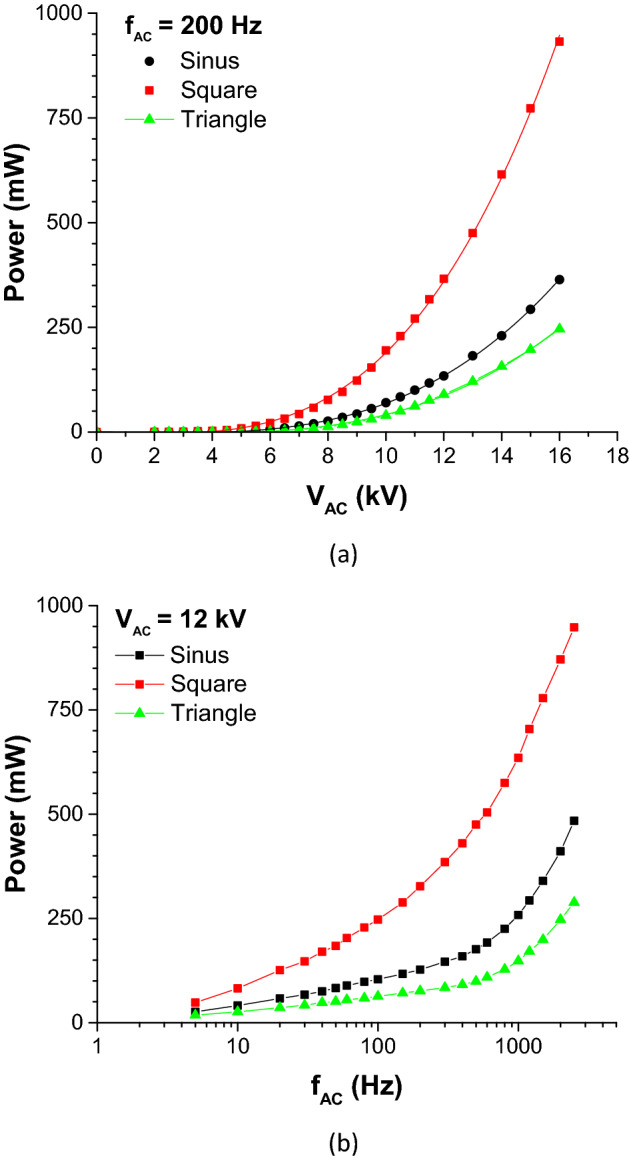
Electrical power consumption versus voltage amplitude V_AC_ for f_AC_ = 200 Hz (**a**) and electrical power consumption versus frequency f_AC_ for V_AC_ = 12 kV (**b**).

Figure 2 shows the electrical power consumption of the DBD for the different waveforms. From Fig. 2a, one can deduce that the power consumption increases with ${V}_{AC}^{3}$ (V_AC_ being the voltage amplitude) and that the square HV consumes about 2.5 times more power than the sine HV, and about four times more than the triangle HV. Furthermore, the power consumption for the sawtooth HV is not represented in this figure for clarity, because it would be perfectly superimposed to the curve of the triangle HV. Figure 2b shows the power consumption versus voltage frequency f_AC_. It can be observed that it increases firstly logarithmically with frequency (up to about 100 Hz, 300 Hz and 500 Hz for the square, sinus and triangle waveforms, respectively), and then, the slope suddenly increases. The last remark is general: the power consumption is comparable in amplitude to the power consumed by DC coronas^27^, as it is weak and limited to about 1 W.

### Time-averaged ionic wind velocity

**Figure 3:**
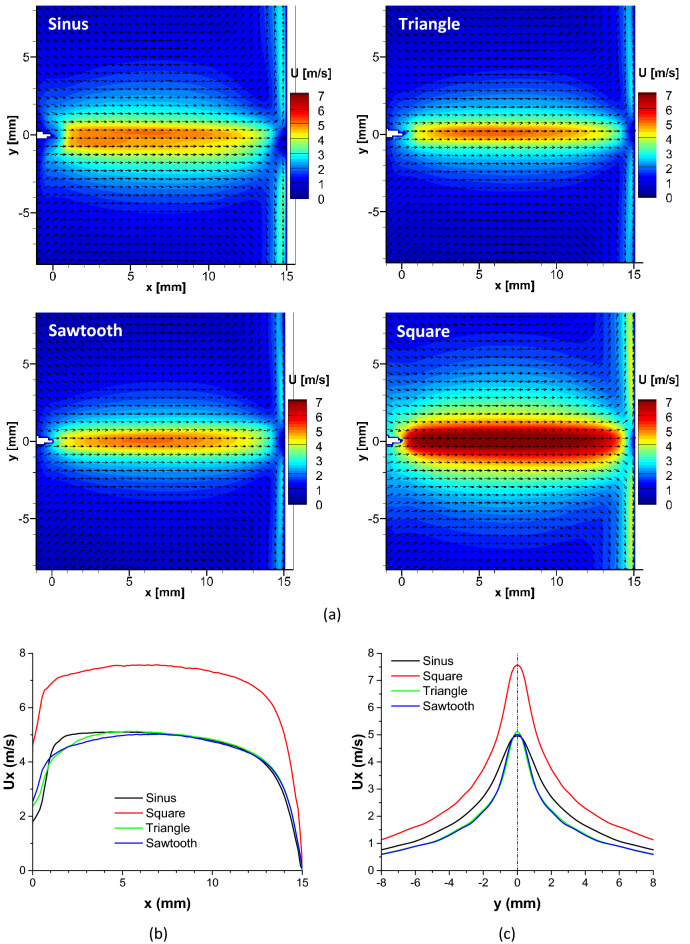
Velocity fields of the produced ionic wind for different voltage waveforms at V_AC_ = 14 kV and f_AC_ = 500 Hz (**a**). Horizontal velocity profiles at y = 0 (**b**) and vertical velocity profiles at x = 5 mm (**c**) for different voltage waveforms (V_AC_ = 14 kV and f_AC_ = 500 Hz).

The coming section focuses on the time-averaged velocity of the ionic wind induced by the needle-to-plate DBD. Figure 3 presents examples of velocity vector fields and velocity profiles for the four HV waveforms (voltage V_AC_ = 14 kV and frequency f_AC_ = 500 Hz). On the velocity fields (Fig. 3a), the needle tip is located at the coordinates (x = 0, y = 0) and the dielectric wall at x = 15 mm, and the field of view (FOV) corresponds to the one showed in Fig. 5b.

Two main remarks can be done. On the one hand, in all the cases, an ionic wind jet starting from the needle tip and flowing toward the opposite electrode can be observed. This jet is parallel to the needle and impacts the dielectric wall at x = 15 mm. On the other hand, the induced jet varies according to the high voltage waveform. Indeed, from these velocity fields, it is clear that the square high voltage results in a faster ionic wind.

To better investigate the influence of the high voltage waveform, Fig. 3b presents profiles of the horizontal component of the velocity U_x_ (in the x direction) plotted from the velocity fields. These profiles are located at y = 0, i.e. in front of the tip, for 0 ≤ x ≤ 15 mm (in practice, this corresponds to the velocity on a straight line from the needle tip to the dielectric wall). Two main points emerge. First, the behavior is always the same, with an increase in the velocity with x before reaching a plateau (with a weak velocity diminution), and then a sudden decrease down to zero when the jet impacts the dielectric wall. Secondly, the square HV results in a faster ionic wind velocity in front of the needle tip.

Figure 3c presents profiles of the horizontal component U_x_ of the velocity at x = 5 mm for the four waveforms. Three main remarks can be made. First, the triangle and sawtooth high voltages result in quasi-similar profiles. This result is interesting, since the electrical power consumption of these two waveforms is the same and equal to 220 mW, highlighting that the electro-mechanical effectiveness is similar. With the sine high voltage (power consumption of 335 mW), the maximum velocity in front of the needle tip is similar but the jet is a little wider. Finally, with a power consumption of 910 mW, the square high voltage allows to produce the faster jet. The reason behind this phenomenon is uncertain. In fact, in order to give a precise conclusion, the determination of the value and distribution of the space charge in the inter-electrode gap is required, and there is no manner to perform such measurements with a sufficient spatial resolution. This is a big problem since it is not possible today to make the link between the physics of the discharge and the resulting ionic wind, because the local electroaerodynamic force that induces the ionic wind is equal to the electric field multiplied by the local space charge.

### Time-resolved ionic wind velocity

**Figure 4:**
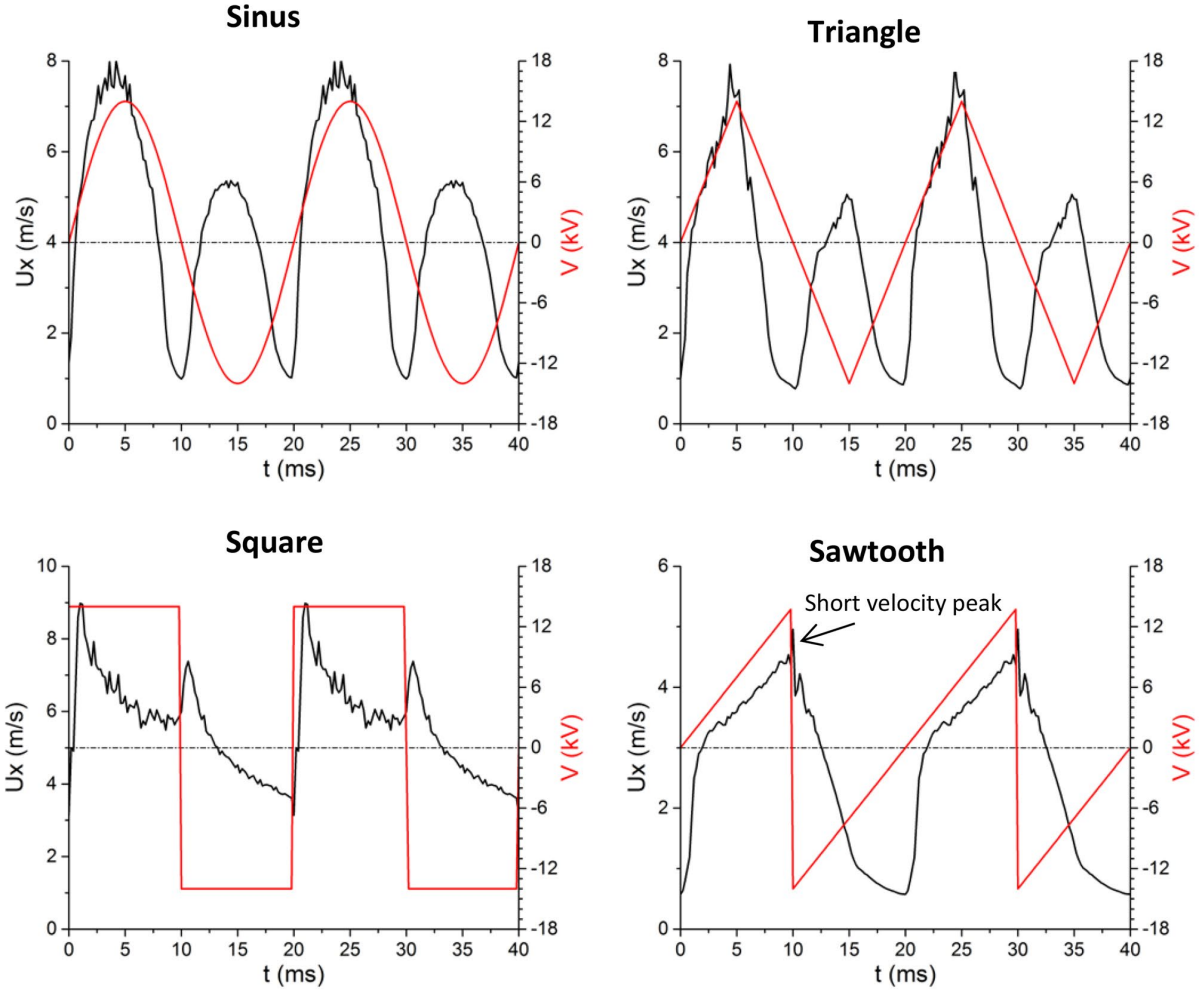
Horizontal component of the instantaneous velocity U_x_ at y = 0 and x = 1.5 mm versus time for the four different voltage waveforms (V_AC_ = 14 kV and f_AC_ = 50 Hz).

After observing the time-averaged velocity of the ionic wind velocity, the study now focuses on its behavior versus time in its periodic and steady state, i.e. a few milliseconds after the voltage is switched on. Figure 4 presents the horizontal component of the velocity U_x_ at y = 0 and x = 1.5 mm, i.e. in front of the needle and spaced by 1.5 mm with the tip, during two cycles (for voltage amplitude V_AC_ = 14 kV, voltage frequency f_AC_ = 50 Hz, then a cycle duration of 20 ms). We can observe that the time behavior of the ionic wind velocity fully depends on the high voltage waveform.

In the case of the sine HV, there are two velocity bumps per cycle; one during the positive half-cycle and the other one during the negative one. In fact, from t = 0, the velocity increases up to a maximum value equal to about 8 m/s. This velocity rise is due to the positive streamer discharge. When the latter ends, the velocity starts to decrease down to about 1 m/s, because there is no more electroaerodynamic force close to the tip. When the negative glow discharge switches on, a second velocity rise takes place. However, the maximum velocity reached during the negative discharge is smaller than the one of the positive discharge. This is in agreement with previous studies, which highlighted that the ionic wind produced by a positive DC corona is stronger than the one of a negative DC corona^25^. In the case of the triangle high voltage, the time behavior of the velocity is slightly different, but there are also two velocity bumps per cycle. That is consistent because the two high voltage waveforms are very similar.

When the discharge is powered by a square high voltage, the time behavior is fully different. At the beginning of the positive half-cycle (t = 0 and 20 ms), there is a sudden velocity augmentation, from less than 4 up to 9 m/s. This strong ionic wind, which starts at the tip of the needle, may be explained by the fact that the electric field is strengthened when the polarity is reversed, due to the many negative ions remaining from the previous negative half-cycle at the dielectric wall. After that, the velocity decreases monotonously until the end of the positive half-cycle. When the voltage v_AC_(t) becomes negative, there is a second velocity bump, but it is weaker than the one occurring at the beginning of the positive half-cycle. One more time, this sudden velocity augmentation is due to the positive ions remaining on the surface of the dielectric, leading to a strengthening of the electric field around the needle tip. In fact, the time behavior of the ionic wind when the discharge is powered by a square high voltage is completely different compared to the two previous ones, because the velocity increases do not occur during the positive and negative discharges but when the polarity is reversed.

In the case of the sawtooth high voltage, the behavior is again very different. During the positive half-cycle (0 ≤ t ≤ 10 ms), the velocity increases gradually. This is due to the positive streamer discharge that is more intense as the voltage rises (see Fig. 1). When the voltage becomes negative, there is a very short velocity peak due to the polarity inversion, and then the velocity decreases rapidly down to 0.6 m/s. This shows that the negative discharge occurring during the negative half-cycle of the triangle waveform is not effective in ionic wind production, with a decay of the velocity higher than the one observed during the negative half-cycle of the square high voltage.

To conclude this article, four main remarks can be drawn. First, the fact that the time-averaged ionic wind velocity is higher with the square high voltage (see Fig. 3c) is certainly due to the positive streamer discharge, since the ionic wind velocity ranges between 9 and 6 m/s during the positive half-cycle when it is limited to 8 m/s for the three others waveforms (see Fig. 4). This is due to the strengthening of the electric field and therefore the strong positive space charge occurring during the rising edge of the voltage. Secondly, although this is not represented here, when approaching the plate (higher x values), the time behavior of the velocity is similar to the one of Fig. 4 but with a time shift. Third, similar temporal behaviors are observed for frequency values f_AC_ up to about 500 Hz. For higher frequencies, the positive discharge takes over the negative one, and from 2000 Hz, there is only one single flow acceleration per voltage cycle.

Finally, the last remark is an observation without answer today. With the volume needle-to-plate discharges reported in the present paper, it is clear that the positive streamer discharge is more efficient in velocity production than the negative discharge, whereas the contrary is observed in the case of surface dielectric barrier discharges taking place at the surface of a dielectric^16^. This result is surprising and it shows that the electroaerodynamic phenomena occurring in such discharges are far from being known and understood. In the future, we will have to try to better understand the physics of these discharges, and especially the interactions that exist between the streamers, the wall of the dielectric material and the surrounding gas, in order to be able to determine the distribution of electrical charges in space and time.

## Methods

**Figure 5:**
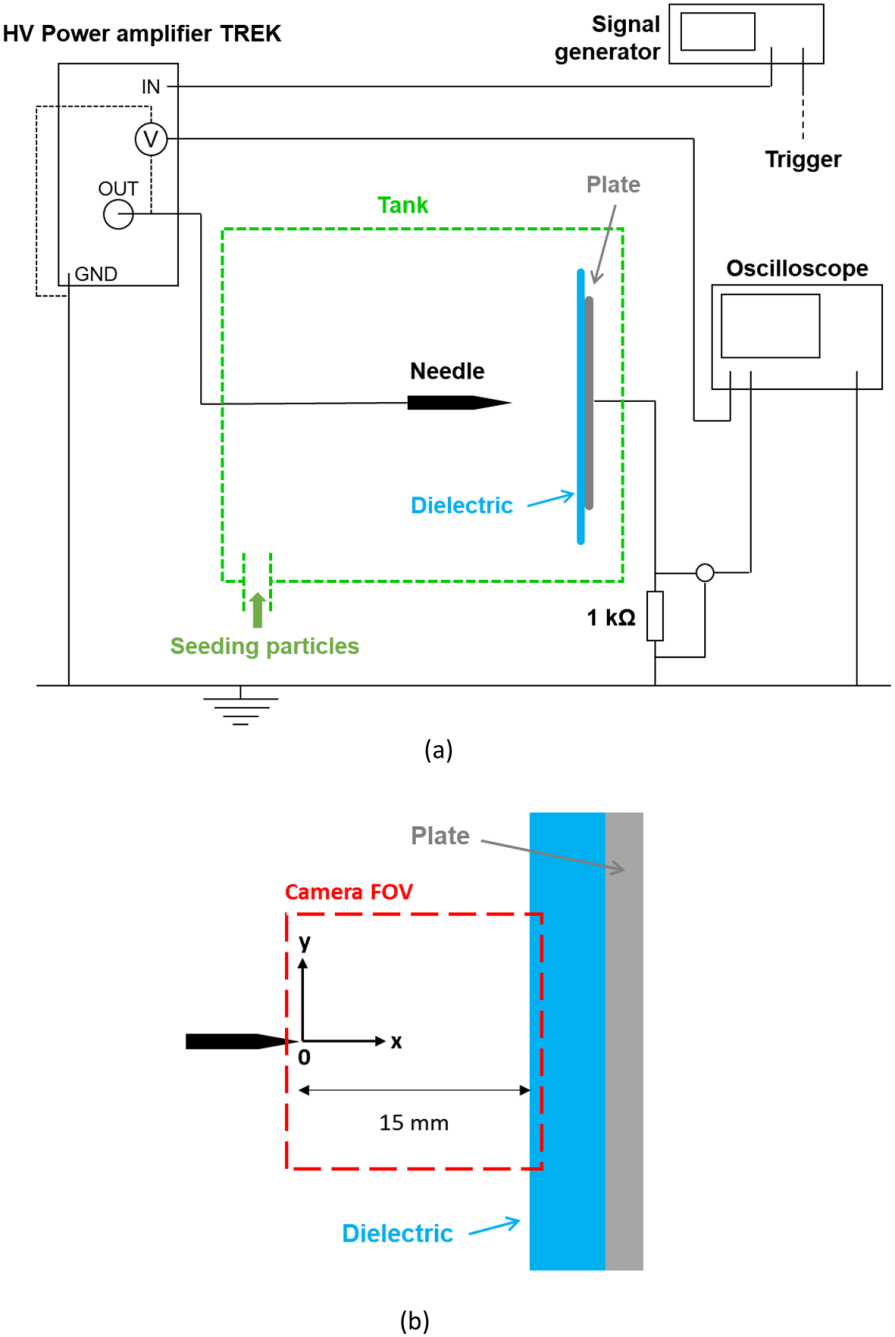
Experimental setup : wide view with the electrical circuit (**a**) and zoomed view of the point-to-plate design with the camera field of view (**b**).

Figure 5A presents the experimental setup with the electrical circuit, and Fig. 5b shows a close-up view of the electrode design. As indicated previously, an AC high voltage needs to be applied between a needle and a grounded plate electrode to ignite the discharge. The needle is in a horizontal position and the plate electrode is placed perpendicularly to the needle. The needle is made of tungsten and its tip has a curvature radius of about 100 µm, when the grounded electrode is a stainless steel disk of 80 mm in diameter, as in Defoort et al.^27^. However, here, the plate electrode is covered with a 5-mm thick dielectric plate made of Polymethyl Methacrylate (PMMA, blue shape in Fig. 5). The distance between the needle tip and the wall of the dielectric plate is equal to 15 mm (Fig. 5b).

The needle is connected to a high voltage amplifier (Trek 30 kV/40 mA, slew rate equal to 600 V/µs) that amplifies a low voltage supplied by a signal generator (Lecroy WaveStation 3082) and that hence provides the high voltage v_AC_(t). The discharge current versus time i(t) is deduced from the measurement of the voltage across a 1 kΩ resistor connected between the plate and electric ground with a voltage probe (Lecroy PP018, 500 MHz, 10 pF). The signals i(t) and v_AC_(t) are recorded with a high-quality digital oscilloscope (Lecroy HDO6054, 12 bits, bandwith of 500 MHz, 10 Gs/s, accuracy of 0.5% full scale). The reproducibility of the measurements was verified.

To characterize the ionic wind produced by the discharge, the setup is exactly the same as in Defoort et al.^27^. A LaVision time-resolved particle imaging velocimetry (PIV) system is used. The discharge design is put into a PMMA tank (30 cm × 80 cm × 40 cm). The air is seeded with dielectric oil droplets (Ondina 915) having a mean diameter equal to 0.3 μm. It has been demonstrated several times that these oil droplets are not significantly impacted by the electrostatic phenomenon. Therefore, it is assumed that they follow well the ionic wind produced by the discharge^25,27^. The light used to illuminate the seeding particles is produced by a 532 nm Nd-YAG laser generator (Continuum Mesa). PIV field images are acquired with a 20 kHz camera (Photron Fastcam SA-Z). The system is operated from a computer with LaVision DaVis software, which also gathers the experimental data. The resulting images have a resolution of 1024 × 1024 pixels, a size of 16.5 mm × 16.5 mm (see the red Field Of View, FOV, Fig. 5b). The time between two successive images is correctly adjusted to obtain the best displacement between two successive images. Its value is around 50 µs. 2000 images are recorded for every measure. The velocity is computed using a cross-correlation algorithm with adaptive multi-passes (LaVision DaVis software), with interrogation windows of 64 × 64 down to 16 × 16 pixels and an overlap set to 50%, leading to a final flow field resolution of one vector every 130 μm (or 7.7 vectors/mm). For the time-averaged velocity fields presented in Fig. 3, the map of the uncertainties of the time-averaged velocity was computed using the linear error propagation technique^30^ and it is always smaller than 0.02 m/s.

## Data Availability

The datasets used and/or analysed during the current study available from the corresponding author on reasonable request.
